# 

**Published:** 1916-10

**Authors:** 


### Meeting of the Southern Medical Association.

On Monday, Tuesday, Wednesday and Thursday, November 13th to 16th, the meeting of the Southern Medical Association will be held in Atlanta, Georgia.

The outstanding feature of the meeting is to be the clinics which will be held each morning from 8 to 10 by visiting physicians from various Southern cities. All the scientific sessions will be held under one roof—the great Auditorium-Armory.

Special entertainment is being provided for the doctors' wives, and a woman's health conference will be held for them.

There will be lots and lots of good things. Don't fail to go and get your share.

